# Fully Assembled Genome Sequence for *Salmonella enterica* subsp. *enterica* Serovar Javiana CFSAN001992

**DOI:** 10.1128/genomeA.00081-13

**Published:** 2013-03-21

**Authors:** Marc W. Allard, Tim Muruvanda, Errol Strain, Ruth Timme, Yan Luo, Charles Wang, Christine E. Keys, Justin Payne, Tony Cooper, Khai Luong, Yi Song, Chen-Shan Chin, Jonas Korlach, Rich J. Roberts, Peter Evans, Steven M. Musser, Eric W. Brown

**Affiliations:** a Office of Regulatory Science, Center for Food Safety & Applied Nutrition, U.S. Food & Drug Administration, College Park, Maryland, USA; b Office of Food Defense, Communications and Emergency Response, Center for Food Safety & Applied Nutrition, U.S. Food & Drug Administration, College Park, Maryland, USA; c Pacific Biosciences, Menlo Park, California, USA; d New England Biolabs, Inc., Ipswich, Massachusetts, USA

## Abstract

We report a closed genome of *Salmonella enterica* subsp. *enterica* serovar Javiana (*S*. Javiana). This serotype is a common food-borne pathogen and is often associated with fresh-cut produce. Complete (finished) genome assemblies will support pilot studies testing the utility of next-generation sequencing (NGS) technologies in public health laboratories.

## GENOME ANNOUNCEMENT

*Salmonella enterica* subsp. *enterica* serovar Javiana (*S*. Javiana) is one of the top five most common serotypes of *Salmonella* associated with fresh-cut produce, with an average of 11 clusters per year (2008 to 2012) detected by the PulseNet Network. This serotype has been involved in several produce-related outbreak investigations over the past decade, including for commodities such as cantaloupes, green onions, and tomatoes. It is also frequently recovered from the growing environments of these products. In October 2012, an outbreak of *S*. Javiana (PulseNet cluster 1210MLJGG01, pulsed-field gel electrophoresis [PFGE] pattern JGGX01.0500) was determined likely to be produce-related, but a specific commodity responsible for the illnesses was not determined. One clinical isolate (AZ_PI12305015) of this cluster was chosen for complete sequencing and assembly. We believe that the availability of whole-genome sequences and a large reference database will provide the discriminatory power needed to facilitate outbreak cluster detection and source tracking (1, 2). Currently, there is only one *S*. Javiana genome available in GenBank (accession no. ABEH00000000).

DNA was isolated from pure culture using Qiagen DNeasy blood & tissue kit (Qiagen Inc., Valencia, CA). Genome sequencing was performed using Pacific Biosciences RS sequencing technology (Pacific Biosciences, Menlo Park, CA), achieving >20× average genome coverage. The sample was prepared as a 10-kb insert library and was sequenced on 8 single-molecule real-time (SMRT) cells. *De novo* assemblies were created for the genome using the DevNet hierarchical genome assembly process (HGAP)/Quiver software package >4.5 kb 0.8QV (http://www.smrtcommunity.com/Share/Code?id=a1q70000000H2qRAAS), followed by Minimus2, and polished with Quiver to yield a single chromosomal contig and two mobile elements. The complete process from isolate to finished genomic sequence took less than 1 week. The mobile elements are single contigs but do not have overlapping sequences at their ends, suggesting one or more gaps. The genomes were annotated with the NCBI (National Center for Biotechnology Information) Prokaryotic Genomes Automatic Annotation Pipeline (http://www.ncbi.nlm.nih.gov/genomes/static/Pipeline.html). The methylome was determined using the kinetic score distributions to detect the presence of ^m6^A methyltransferases (MTases) (3, 4).

Comparative genomic analyses of these data will be included in future publications. This will include descriptions of the 4 active ^m6^A methytransferases observed in *S*. Javiana. The methylome of this strain was distinct from other strains analyzed thus far in Rebase (http://rebase.neb.com/cgi-bin/pacbiolist) (5).

This data release is a contribution toward the efforts of the 100K Pathogen Genome Project consortium. The FDA, along with Agilent Technologies, University of California at Davis, and many other federal and private partners, will sequence 100,000 pathogen genomes over the next 5 years (http://100kgenome.vetmed.ucdavis.edu) and will include genome closure of many of these isolates and their mobile elements. The product of this enormous effort will be a public molecular epidemiology reference database useful for designing pathogen detection assays, providing the evolutionary context for outbreaks, and many other applications yet to be realized (6, 7). The NGS (next-generation sequencing) federal and state public health laboratory aspect of the public database will be housed at the NCBI, in Bethesda, MD, under bioproject no. 183844 (http://www.ncbi.nlm.nih.gov/bioproject/183844) and known as the GenomeTrakr database.

### Nucleotide sequence accession numbers.

Genome sequences of *S*. Javiana and its mobile elements are available in DDBJ/EMBL/GenBank under bioproject no. 184141 and the GenBank accession no. CP004026, CP004027, and CP004028 at NCBI. Kinetic information is deposited in Gene Expression Omnibus (GEO) (GSE45178).
