# Fully Assembled Genome Sequence for *Salmonella enterica* subsp. *enterica* Serovar Javiana CFSAN001992

**DOI:** 10.1128/genomeA.00293-14

**Published:** 2014-04-03

**Authors:** Marc W. Allard, Tim Muruvanda, Errol Strain, Ruth Timme, Yan Luo, Charles Wang, Christine E. Keys, Justin Payne, Tony Cooper, Khai Luong, Yi Song, Chen-Shan Chin, Jonas Korlach, Richard J. Roberts, Peter Evans, Steven M. Musser, Eric W. Brown

**Affiliations:** a Office of Regulatory Science, Center for Food Safety & Applied Nutrition, U.S. Food & Drug Administration, College Park, Maryland, USA; b Office of Food Defense, Communications and Emergency Response, Center for Food Safety & Applied Nutrition, U.S. Food & Drug Administration, College Park, Maryland, USA; c Pacific Biosciences, Menlo Park, California, USA; d New England Biolabs, Inc., Ipswich, Massachusetts, USA

## AUTHOR CORRECTION

Volume 1, no. 2, e00081-13, 2013. Page 1: The article byline should read as given above.

Page 2: The following should be added to the Acknowledgments. “Research reported in this publication was supported by the Small Business Innovation Research Program (NIGMS) of the National Institutes of Health under award number R44GM105125 to R.J.R.

The content is solely the responsibility of the authors and does not necessarily represent the official views of the National Institutes of Health.”

